# Impact of interaction style and degree on the evolution of cooperation on Barabási–Albert scale-free network

**DOI:** 10.1371/journal.pone.0182523

**Published:** 2017-08-14

**Authors:** Fengjie Xie, Jing Shi, Jun Lin

**Affiliations:** 1 Department of Information Management, College of Economics and Management, Xi’an University of Posts and Telecommunications, Xi’an, Shaan Xi, China; 2 Department of Mechanical and Materials Engineering, College of Engineering & Applied Science, University of Cincinnati, Cincinnati, Ohio, United States of America; 3 Department of Management Science, School of Management, Xi’an Jiaotong University, Xi’an, Shaan Xi, China; Beihang University, CHINA

## Abstract

In this work, we study an evolutionary prisoner’s dilemma game (PDG) on Barabási–Albert scale-free networks with limited player interactions, and explore the effect of interaction style and degree on cooperation. The results show that high-degree preference interaction, namely the most applicable interaction in the real world, is less beneficial for emergence of cooperation on scale-free networks than random interaction. Besides, cooperation on scale-free networks is enhanced with the increase of interaction degree regardless whether the interaction is high-degree preference or random. If the interaction degree is very low, the cooperation level on scale-free networks is much lower than that on regular ring networks, which is against the common belief that scale-free networks must be more beneficial for cooperation. Our analysis indicates that the interaction relations, the strategy and the game payoff of high-connectivity players play important roles in the evolution of cooperation on scale-free networks. A certain number of interactions are necessary for scale-free networks to exhibit strong capability of facilitating cooperation. Our work provides important insight for members on how to interact with others in a social organization.

## Introduction

Understanding the emergence of cooperation among selfish individuals is one of the central topics in evolutionary game theory [1].This problem remains a challenge in a social dilemma situation because the evolutionary equilibrium predicted by game theory is defection, not cooperation. Many natural and social scientists have investigated the possible mechanisms for the emergence of cooperation in a popular social dilemma, i.e., prisoner’s dilemma game (PDG). Several effective mechanisms have been identified, including kin selection, group selection, direct reciprocity, reputation, and spatial structure [2].

Spatial structure means that some individuals interact more often than others. One approach of capturing this effect is the evolutionary graph theory [3], which allows us to study how spatial structure or social network affects the evolution of cooperation. Nowak and May first proposed this mechanism in 1992 [4]. In their pioneering work, players occupy the nodes of a square lattice, play PDG with four neighbors, collect an aggregate payoff, and imitate the strategy of the most successful neighbor. Their simulation results show that cooperation can be obtained in PDG, and the clusters formed by cooperators on a square lattice are crucial for prevailing cooperative behaviors. Following this work, a wealth of studies provides additional evidence for the positive effect of spatial lattices on cooperation in PDG, but the spatial effects are closely related to lattice minutiae [5,6], social diversity [7–9], teaching activity [10,11], variable investment [12], heterogeneous coupling [13], and population density [14].

In a spatial lattice, a player can interact with his/her neighbors in some type of array, and each player has the same number of neighbors. However, social interaction situations are rarely described by such an extreme style. Some more compelling network patterns, such as small-world networks [15,16] and scale-free networks [17], are used to characterize the interactions between social individuals. The studies about the evolution of PDG on these networks [18–21] show that cooperation depends heavily on the interaction pattern defined by different network structures. Among these studies, a well-known conclusion is that scale-free networks produce a much higher cooperation level than regular ring networks and provide a unifying framework for the emergence of cooperation [21]. Based on that, a large number of studies have addressed various aspects related to the evolution of PDG on scale-free networks.

Firstly, the degree distribution of scale-free networks, which follows a power-law pattern and exhibits higher level of structural heterogeneity than that of other networks (such as random networks, single-scale networks and broad-scale networks), is recognized as a prominent factor for cooperation in evolutionary game dynamics [22]. Furthermore, it is found that the variance of the degree distribution affects the evolution of cooperation, and there exists an appropriate value of the variance, at which cooperation is optimal [23].

Secondly, since social networks have non-trivial clustering [15,16], assortativity mixing [24,25] and community structure [26,27], which differ from most other types of networks (such as technological networks and biological networks), the effect of these structural properties of scale-free networks on cooperation have also been widely explored. It is found that high clustering and obvious community structure of scale-free networks have positive effect on cooperation [28–31], but assortativity mixing of scale-free networks hinders the evolution of cooperation [32].

Thirdly, the payoff mechanism, which describes how each player’s payoff is calculated in each round game, influences the evolution of cooperation. If the mechanism changes from accumulated payoff to average payoff, the outstanding ability of scale-free networks to facilitate cooperation found in the work [21] deteriorates continuously, eventually collapsing with the results obtained on regular graphs [33]. While an asymmetric payoff mechanism is introduced and the hub nodes are favored in the payoff matrix, the cooperation is further promoted on scale-free networks [34]. Under the measurement of average payoffs, cooperation is sometimes inhibited and sometimes enhanced on the scale-free networks, with respect to the cases on the random regular networks and regular lattices [35].

Lastly, some other mechanisms also have non-trivial influences on cooperation in scale-free networks. Under a preferential selection mechanism, the level of cooperation is greatly influenced by the initial strategies of hub nodes [36]. While error and attach mechanism are introduced, cooperation is extremely robust against random deletion of nodes, but declines quickly if nodes with the maximal degree are targeted [37]. Social diversity [38], tie weight [39,40], conformity [41], strategy update rules [42,43], punishment [44] and emotion [45] have shown to reinforce the cooperation in scale-free networks.

It is worth mentioning that under different payoff mechanisms, the effects of scale-free networks on cooperation are different accordingly [21,33–35]. Average payoff scheme emphasizes that everyone in the real world has limited time and energy, and so an individual with numerous connections is unlikely to interact with all neighbors in every round of game. By dividing the accumulated payoff of a player by his/her number of neighbors, average payoff scheme measures the average level of payoff that a player gets from one interaction. It reflects an implicit assumption that all players have the same capability in interacting with others, i.e., obtaining payoff from one interaction in every round of game. This conflicts with the actual situation in the real world where some people are more adept at interactions than most others. Besides, the theoretical results obtained by Wu et al. [35] show that under the measurement of average payoff, game payoffs of high-connectivity players are far smaller than those of low-connectivity players for some parameter conditions. This is unreasonable, because high-connectivity players in social organizations are generally the well-known persons or leaders, whose payoffs are universally higher than ordinary people. Thus, we argue that average payoff mechanism eliminates too much benefit of having a high degree in scale-free networks. Conversely, accumulated payoff is more reasonable for payoff measurement in scale-free networks, as long as we can explicitly include time or energy limitation of players in simulation model, i.e., there should be a restriction on player interactions in every round of the game.

Note that such a restriction on player interactions is briefly attempted for cooperation on scale-free networks in literature [46]. In that work, the players interact with limited neighbors randomly. However, random interaction only accounts for a small fraction of interaction situations in the real world. In most cases, we interact with each other with preference. To this end, we introduce a realistic interaction style, i.e., high-degree preference interaction, which is opposite to the random interaction applied in the literature [46] and is rooted in obtaining more benefits from the individuals who have more connections and resources. Equally important, since the overwhelming advantage of scale-free networks against regular ring networks found in the work [21] is based on full interactions, it is intriguing to investigate whether this kind of advantage still exist while limited interaction is considered. This part is also not considered in the literature [46]. Our work makes significant contributions by revealing the importance of interaction style and degree for cooperation, which provides us insight into how to interact with others in a social organization especially when time and energy are limited.

The remainder of this paper is organized as follows. Section 2 describes the model. Section 3 presents the simulation results and the analysis. Section 4 summarizes our findings and concludes the paper.

## Model

Scale-free networks are widespread in the real world and are characterized by a degree distribution that decays as a power-law. A theoretical scale-free network can be generated by the Barabási-Albert model [17]. Two important processes, namely growth and preferential attachment, are integrated into the model. The growth process starts with a small number (*m*_0_) of nodes, and at every time step, a new node with *m*(≤ *m*_0_) edges is linked to *m* different nodes already present in the system. For the preferential attachment process, the probability Π(*k*_*i*_) that a new node will be connected to node *i* depends on the degree *k*_*i*_ of node *i*, such that

$$\Pi (k_{i})=\frac{k_{i}}{\sum_{i}k_{i}}.$$ (1)

Prisoner’s dilemma game (PDG) describes the interactions of two players with two possible behavioral options: cooperate (C-strategy) or defect (D-strategy). Two players must simultaneously decide whether to cooperate or defect. They both receive *R* upon mutual cooperation and *P* upon mutual defection. A defector exploiting a cooperator gets an amount *T* and the exploited cooperator receives *S*, such that *T*>*R*>*P*>*S* and 2*R* > *T* + *S*.

**Fig 1:**
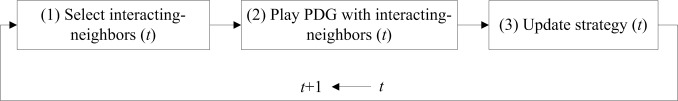
The interacting process of players.

Each player in PDG occupies a node of the scale-free network generated by the Barabási-Albert model. If player *i* has an edge with player *j* in the network, then player *j* is a neighbor of player *i* and vice versa. Three important procedures are implemented by each player at each game time *t*: select interacting-neighbors; play PDG with interacting-neighbors; update strategy. The process can be summarized in Fig 1.

### Select interacting-neighbors

Players sequentially select interacting-neighbors based on their degrees. The higher the degree a player has in the scale-free network, the higher the priority he/she has for selecting interacting-neighbors. For the players with the same degree, the priority among them is assigned randomly. Such a priority for high-connectivity individuals can be observed universally in the real world.

Player *i* selects his/her interacting-neighbors using two possible rules, high-degree preference rule and random rule. Under the high-degree preference rule, player *i* prefers interacting with high-connectivity neighbors. This preference is rooted in obtaining more benefits from the individuals who have more connections and resources. Then, the probability Π(*k*_*j*_) that player *i* will propose an interaction with neighbor *j* depends on the degree *k*_*j*_ of *j*, such that

$$\Pi (k_{j})=\frac{k_{j}}{\sum_{j\in \Omega_{i}}k_{j}}.$$ (2)

where Ω_*i*_ is the neighbor set of player *i*. Under the random rule, player *i* treats his/her neighbors equally and will propose an interaction with neighbor *j* randomly. This rule can be applied if the interactions among players are exogenously determined, and is a valuable comparison of high-degree preference interaction.

If player *i* has less than *W* interacting-neighbors, he/she can propose an interaction with a neighbor *j* based on high-degree preference rule or random rule. Given that player *i* proposes an interaction with the neighbor *j*, a reciprocal interaction relation between them will be built only if neighbor *j* also has less than *W* interacting-neighbors. In this situation, neighbor *j* becomes the ‘interacting-neighbor’ of player *i* at game time *t*, and vice versa.

Player *i* tries to find *W* interacting-neighbors at game time *t*. However, if there are not enough potential interacting-neighbors around him/her or if all potential interacting-neighbors have already been “reserved” by other interactions, player *i* has to stop selecting, and therefore he/she has less than *W* interacting-neighbors or no interacting-neighbors at game time *t*. This means that if a player has a degree larger than *W*, some of his/her neighbors inevitably have no interactions with the player. Such an interaction situation corresponds to some actual cases in the real world. For example, during a given period of time, such as one day, an individual who has many friends can only meet with some of them, and a researcher who has many collaborators can only work with several of them. Here, *W* is referred to as the “upper-bound” and represents the limited interaction degree of players.

### Play PDG with interacting-neighbors

After all players complete the procedure of selecting interacting-neighbors, the PDG at time *t* begins among players who have reciprocal interaction relations. Player *i* uses the same strategy in all his/her interactions. Let *x*_*i*,*t*_ denote player *i*’s strategy at game time *t*, *x*_*i*,*t*_ = (1,0) and *x*_*i*,*t*_ = (0,1) represent C-strategy and D-strategy, respectively. The payoff *U*_*i*,*t*_ of player *i* at game time *t* is then obtained by summing the payoffs from all his/her interacting-neighbors and can be expressed as

$$U_{i,t}=\sum_{j\in \Omega_{i,t}^{a}}x_{i,t}\cdot A\cdot x_{j,t}^{'}$$ (3)

where $\Omega_{i,t}^{a}$ is the interacting-neighbor set of player *i* at game time *t*, $A=\left[\begin{array}{cc} R & S\\ T & P \end{array}\right]$ is the payoff matrix of PDG, and $x_{j,t}^{'}$ is the transposition of *x*_*j*,*t*_.

### Update strategy

After all players obtained their game payoff, the strategy updating begins. All players update strategy synchronously. While player *i* updates his/her strategy, he/she first randomly selects an interacting-neighbor *j* from set $\Omega_{i,t}^{a}$. Then, player *i* compares the payoffs of *j* and himself/herself. If *U*_*j*,*t*_ > *U*_*i*,*t*_, he/she will adopt the strategy of *j* at the next game time *t* + 1 with the probability given by

$$p_{i\leftarrow j}=(U_{j,t}-U_{i,t})/Tk_{>}$$ (4)

where $k_{>}=\max(n_{\Omega_{i,t}^{a}},n_{\Omega_{j,t}^{a}})$, $n_{\Omega_{i,t}^{a}}$ and $n_{\Omega_{j,t}^{a}}$ are the number of interacting-neighbors in set $\Omega_{i,t}^{a}$ and $\Omega_{j,t}^{a}$, respectively. Otherwise, if *U*_*j*,*t*_ ≤ *U*_*i*,*t*_, player *i* keeps his/her strategy at the next game time *t* + 1, i.e., *x*_*i*,*t*+1_ = *x*_*i*,*t*_. If a player has no interacting-neighbors at game time *t* (i.e., $n_{\Omega_{i,t}^{a}}=0$), he/she also keeps his/her strategy. After all players have updated their strategies, the next game time *t* + 1 begins.

## Simulation experiment

### Simulation parameter settings

The current model is conducted on the scale-free networks with parameters *m* = *m*_0_ = 2. Population sizes *N* = 1000,10000 and upper-bounds *W* = 4,6,8,12,16,32 are examined. Following common practice in literatures [2, 21,46], PDG parameters are set as *R* = 1, *P* = *S* = 0, *T* = *b*, which have the same qualitative properties as the typical PDG with *P* > *S* when an evolutionary PDG is played on networks [3]. The only one free parameter *b* (2>*b*>1) represents the advantage of defectors over cooperators and is generally referred to as ‘defect temptation’.

The initial strategies (cooperation or defection) are randomly assigned among players with equal probability, and so the frequency of cooperator *f*_*C*_(*t*) in the whole population at game time *t* for the initial state is about 0.5 (i.e., *f*_*C*_(0) ≈ 0.5). The interaction process shown in Fig 1 proceeds for 11000 game time steps (i.e., the simulation time *t* = 11000), and the equilibrium frequency of cooperators *f*_*C*_ is obtained by averaging the last 1000 time steps after a transient time of 10000, that is, $f_{C}=\frac{1}{1000}\sum_{t=10001}^{11000}f_{C}(t)$. Using Monte Carlo (MC) simulations we calculate the average density of cooperators *P*_*C*_ as a function of the defect temptation *b* in the equilibrium state. Each data point of *P*_*C*_ is the average of 20 equilibrium frequencies of cooperators *f*_*C*_ for the same simulation parameter.

### Results and analysis

**Fig 2:**
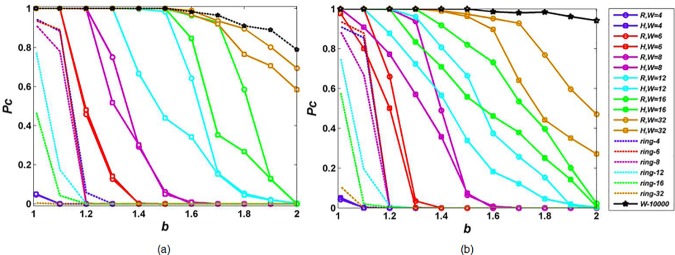
**Density of cooperators** *P*_*C*_ **on scale-free networks as a function of defection temptation *b* for different upper-bounds *W* under population size (a) *N* = 1000 and (b) *N* = 10000.** *R* and *H* correspond to random interaction and high-degree interaction, respectively. The result on corresponding regular ring-*W* networks is denoted by the dotted line with the same color.

**Fig 3:**
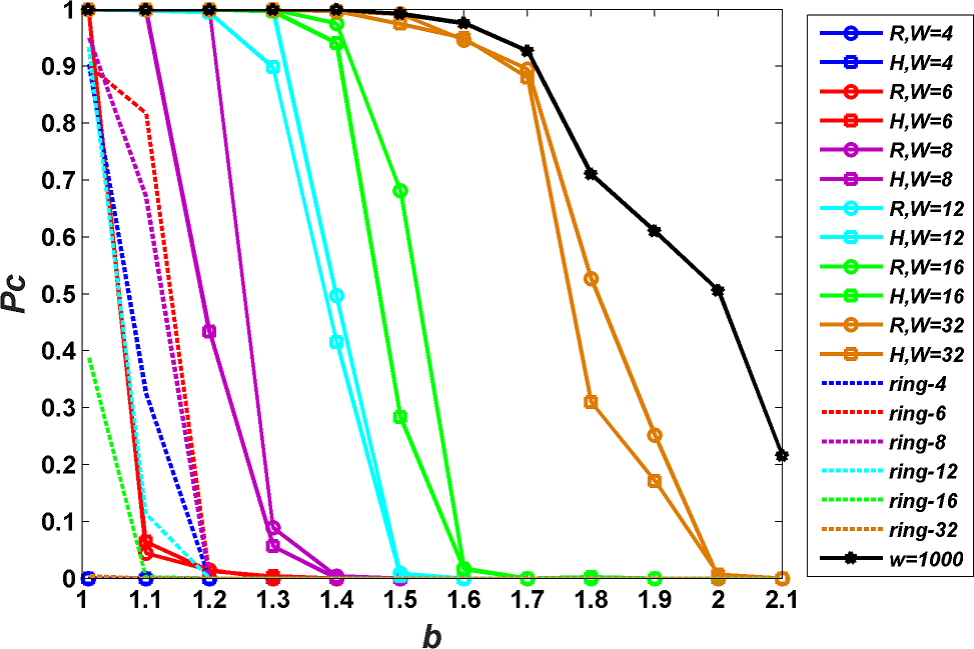
Density of cooperators *P*_*C*_ on scale-free networks as a function of defection temptation *b* for different upper-bounds *W* under a standard PDG parameter combination *R* = 1, *P* = 0, *S* = −0.1,2.1 > *b* > 1. Population size is set as *N* = 1000. *R* and *H* correspond to random interaction and high-degree interaction, respectively. The result on corresponding regular ring-*W* networks is denoted by the dotted line with the same color.

Fig 2 shows the density of cooperators *P*_*C*_ on scale-free networks for upper-bounds *W* = 4,6,8,12,16,32 under two interaction styles (random and high degree preference) and two population sizes (*N* = 1000,10000). In order to investigate whether the overwhelming advantage of scale-free networks against regular ring networks found in literature [21] still exist while limited interaction is considered, the game dynamics defined by Eq (3) and Eq (4) is run on a corresponding regular ring network where all players have a degree of *W*, which is called ring-*W* network here. As a comparison, the case where there is no actual restriction on interactions is examined by setting upper-bound as *W* = *N*, because no player has a degree higher than population size. Besides, we checked a standard PDG parameter combination *R* = 1, *P* = 0, *S* = −0.1, *T* = *b*(2.1>*b*>1) which satisfies *T*>*R*>*P*>*S* and 2*R*>*T*+*S*. The results are presented in Fig 3.

It can be seen from Figs 2A, 2B and 3 that although the density of cooperators *P*_*C*_ has some trivial differences, it shows the same qualitative properties under different population sizes *N* = 1000,10000 and different prisoner’s dilemma parameter settings (*R* = 1, *P* = *S* = 0, *T* = *b*(2 > *b* > 1) and *R* = 1, *P* = 0, *S* = −0.1,2.1 > *b* > 1). Firstly, the density of cooperators *P*_*C*_ decreases with defection temptation *b*. This is because, a higher value of *b* makes defective behavior more competitive and players are more favorable of defecting. Secondly, the effects of interaction style and interaction degree on cooperation on scale free networks also exhibit the same characteristics. For a given upper-bound *W*, random interaction is better for the evolution of cooperation compared with high-degree preference interaction. For a given interaction style, upper-bounds *W* have positive effect on cooperation. While the value of upper-bounds *W* is very low (*W* = 4), *P*_*C*_ on scale-free networks is much lower than that on corresponding ring-4 network, which is against the common perception that scale-free networks must be more beneficial for cooperation than regular ring networks. Next, we analyze and discuss the effects of interaction style and interaction degree on cooperation on scale free networks based on the data of Fig 2A.

#### Analysis for the impact of interaction style on cooperation

**Fig 4:**
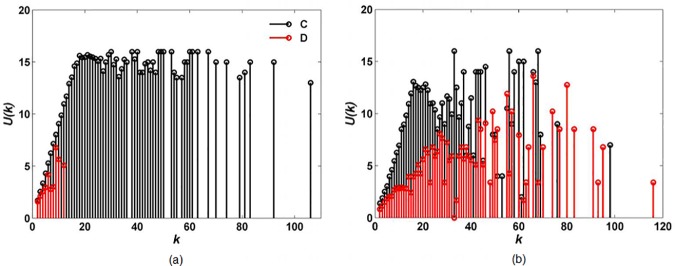
**Average payoff** *U*(*k*) **of a player of degree** *k* **in the stationary regime of evolutionary dynamics under (a) random interaction and (b) high-degree preference interaction.** Cooperator is denoted by C with black and defector by D with red. Defect temptation and upper-bound are set to *b* = 1.7 and *W* = 16, respectively.

To understand the effect of interaction style on cooperation we first examine the average payoff *U*(*k*) of a player of degree *k* in the stationary regime of evolutionary dynamics. For a given realization *l* of initial condition (*W*, *b*, interaction rule), we calculate the final number $n_{l}^{C}(k)$ of cooperators of degree *k* and sum their final payoff $U_{l}^{C}(k)$. The average payoff of a cooperator of degree *k* is then computed as $U^{C}(k)=\sum_{l}U_{l}^{C}(k)/\sum_{l}n_{l}^{C}(k)$. In the same way, the average payoff of a defector of degree *k* is computed as $U^{D}(k)=\sum_{l}U_{l}^{D}(k)/\sum_{l}n_{l}^{D}(k)$. The results for *l* = 20 are provided in Fig 4.

Fig 4A shows that all high-connectivity players cooperate under random interaction and obtain much higher game payoffs than defectors. However, in Fig 4B, a lot of high-connectivity players defect under high-degree preference interaction and the payoffs of them are nearly the same as those of high-connectivity cooperators. This is because, under different interaction styles, the interacting-neighbors of high-connectivity players are different. More specifically, under random interaction, each high-connectivity player selects interacting-neighbors randomly from his/her neighbor set, and so they are surrounded by interacting-neighbors with different degrees. However, under high-degree preference interaction, each high-connectivity player prefers selecting other high-connectivity players. Accordingly, high-connectivity players form a core group where they interact with each other actively. Such an interaction situation can be observed anywhere in the real world, and is similar to the interaction on an assortative network.

**Fig 5:**
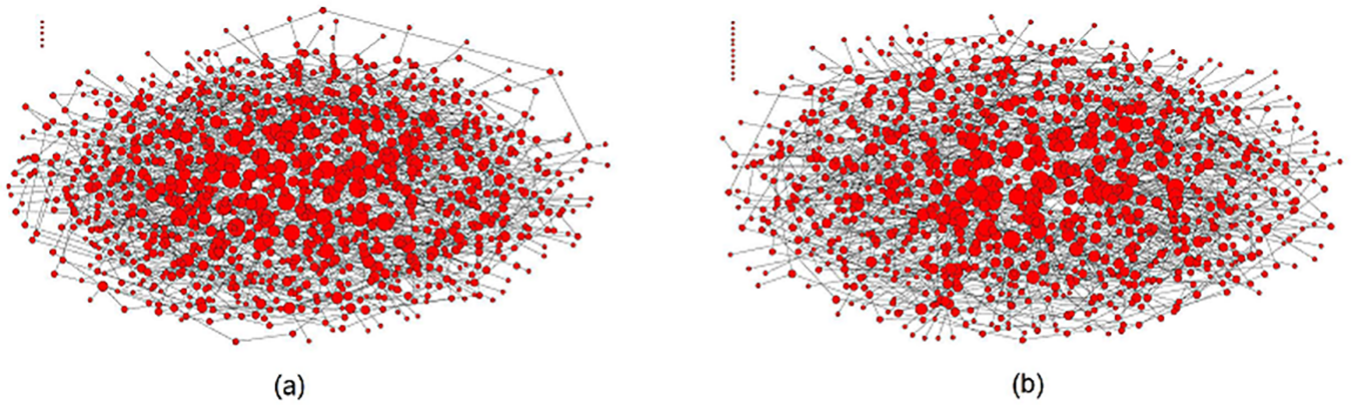
**Interaction relation networks formed by all players in the stationary regime of evolutionary dynamics under (a) random interaction and (b) high-degree preference interaction.** Defect temptation and upper-bound are set to *b* = 1.7 and *W* = 16, respectively.

**Fig 6:**
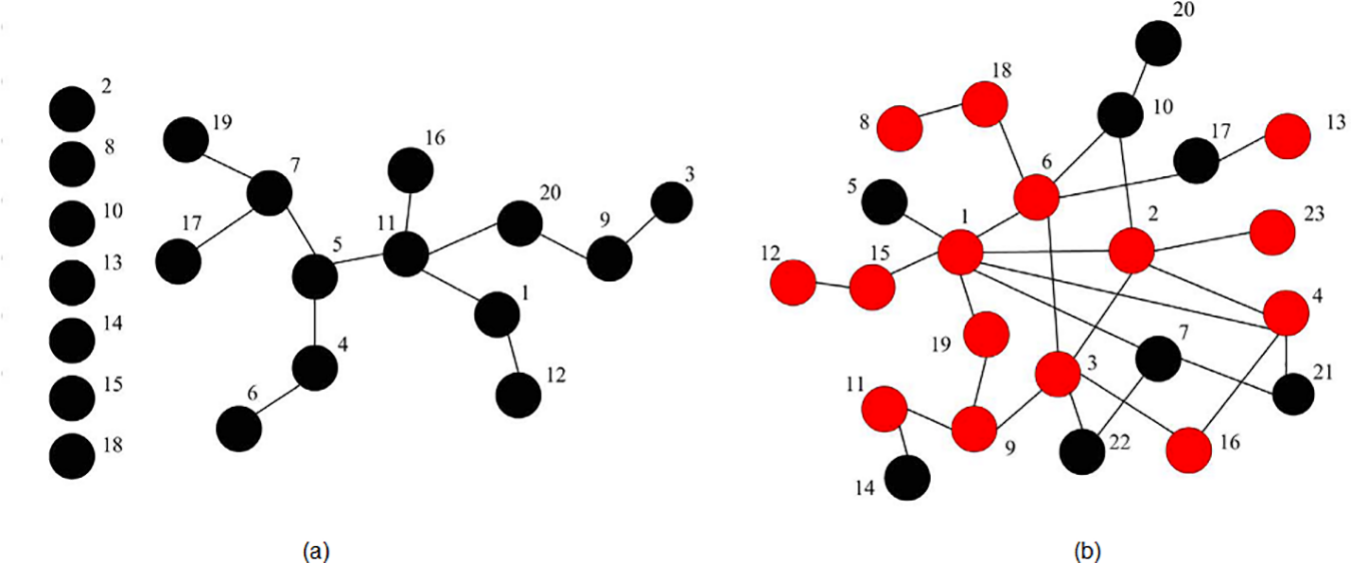
**Interaction relations among high-connectivity players and their strategies in the stationary regime of evolutionary dynamics under (a) random interaction and (b) high-degree preference interaction.** Defect temptation and upper-bound are set to *b* = 1.7 and *W* = 16, respectively. Cooperator is denoted with black node and defector with red node.

Rong et al. [32] indicate that, when players interact on an assortative network, high-connectivity players tend to be interconnected and compose a core group, and then the ability of them to self-sustain the cooperation becomes weaker. Similarly, under high-degree preference interaction, high-connectivity players prefer forming an interacting core group. Then, many high-connectivity players defect other than cooperate. Such a phenomenon is verified by the network presentation in Figs 5 and 6.

Fig 5 shows the interaction relation networks formed by 1000 players in the stationary regime of evolutionary dynamics under two different interaction styles while the upper-bound is set as *W* = 16. In both interaction relation networks, the highest interaction number of players is 16 and the lowest one is 0. It has been explored that there are 20 players and 23 players whose interaction number is 16 in Fig 5A and 5B, respectively, and all of them are the high-connectivity players in the corresponding scale-free networks. Besides, there are 5 players and 11 players whose interaction number is 0 in Fig 5A and 5B, respectively, and all of them are just the neighbors of the players whose interaction number is 16. It seems that there is no significant difference in interaction relation networks under two different interaction styles. However, when we make further exploration on the interactions among the high-connectivity players, i.e., the players whose interaction number is 16 in the stationary regime of evolutionary dynamics, significant difference is found, as shown in Fig 6.

**Fig 7:**
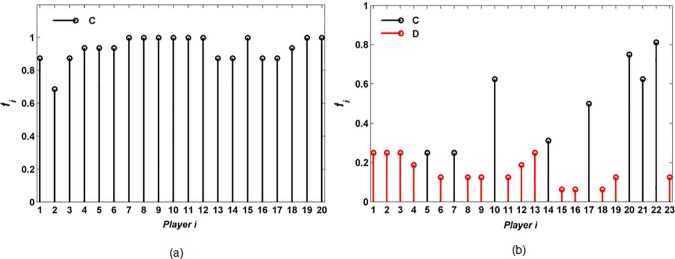
**Frequency of the cooperative interacting-neighbors of high-connectivity players under (a) random interaction and (b) high-degree preference interaction.** Cooperator is denoted by C with black and defector by D with red.

It can be seen in Fig 6A that high-connectivity players 2, 8, 10, 13, 14, 15, and 18 have no actual interactions with others, and all high-connectivity players cooperate. However, in Fig 6B, all high-connectivity players form an interacting core group, and many of them (players 5, 7, 10, 14, 17, 20, 21, and 22) defect. Furthermore, we investigate the frequency of the cooperative interacting-neighbors *f*_*i*_ of each high-connectivity player *i* in Fig 6. The results are shown in Fig 7.

It can be seen in Fig 7A that the frequency of the cooperative interacting-neighbors is very high, which means that most of interacting-neighbors of high-connectivity cooperators are also cooperators. In Fig 7B, the frequency of the cooperative interacting-neighbors of a high-connectivity defector is obviously lower than that of a high-connectivity cooperator, which means that high-connectivity defectors have very strong ability in influencing their interacting-neighbors to select defective behavior.

In sum, the relevant analysis from Figs 4 to 7 indicates that under high-degree preference interaction, the high-connectivity players form an interacting core group. Accordingly, it is easy for a high-connectivity defector to invade the core group and then many high-connectivity players defect other than cooperate. This further influences their interacting-neighbors to choose defective behavior. Inversely, under random interaction, high-connectivity players prefer cooperating and obtain higher game payoff than defectors, which influence their interacting-neighbors to cooperate. As a result, random interaction is better for the evolution of cooperation compared with high-degree preference interaction. Of course, such a difference cannot be observed for all the upper-bounds *W*. This is because, for a very low upper-bounds, such as *W* = 4 or *W* = 6, it is difficult for high-connectivity players to form the interacting core group even though high-degree preference interaction is implemented.

#### Analysis for the impact of interaction degree on cooperation

**Fig 8:**
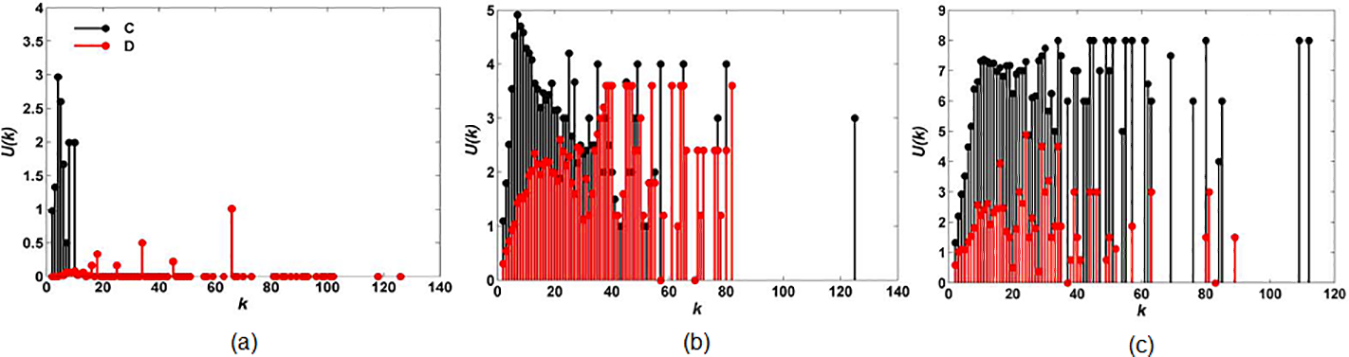
**Average payoff** *U*(*k*) **of a player of degree** *k* **in the stationary regime of evolutionary dynamics under (a)** *W* = 4, *b* = 1.01, **(b)** *W* = 6, *b* = 1.2 **and (c)** *W* = 8, *b* = 1.3. Cooperator is denoted by C with black and defector by D with red.

To understand the effect of interaction degree on cooperation we first examine the average payoff $U^{C}(k)=\sum_{l}U_{l}^{C}(k)/\sum_{l}n_{l}^{C}(k)$ of a cooperator (and $U^{D}(k)=\sum_{l}U_{l}^{D}(k)/\sum_{l}n_{l}^{D}(k)$ of a defector) of degree *k* in the stationary regime of evolutionary dynamics. The results for random interaction rule and *l* = 20 are shown in Fig 8. High-degree interaction rule shows the same qualitative properties as the random case.

For the upper-bound *W* = 4 (Fig 8A), some low-connectivity players whose degrees are no more than *k* = 8 cooperate, but all high-connectivity players defect and obtain no payoff (*U*(*k*) = 0).With the increase of upper-bound (*W* = 6 in Fig 8B and *W* = 8 in Fig 8C), more high-connectivity players cooperate and obtain higher payoff than defective ones, even though defect temptation increases at *b* = 1.2 and *b* = 1.3, respectively.

**Fig 9:**
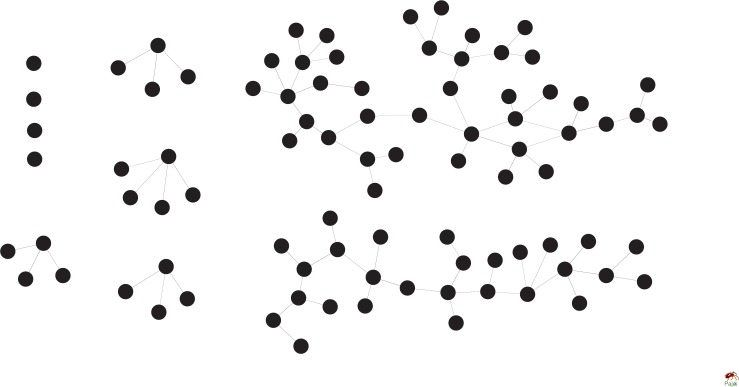
Interaction relations among all cooperators in the stationary regime of evolutionary dynamics under upper-bound *W* = 4. Defect temptation is set to *b* = 1.01.

Although some low-connectivity cooperators Fig 8A obtain higher payoff than others, it is difficult for them to form a big cooperative cluster which is conductive to the evolution of cooperation. Fig 9 shows the interaction relations among all cooperators in the stationary regime of evolutionary dynamics under upper-bound *W* = 4.

It can be seen in Fig 9 that cooperators are separated in different sub-graph, and those in the biggest connected sub-graph only account for 45% of all the cooperators. The reason for such a phenomenon is that, high-connectivity players defect and obtain no payoff under upper-bound *W* = 4, which leads to the fact that they have no influence on the strategy decision of their interacting-neighbors. In this situation, high-connectivity players act as being ‘deleted’ during the strategy evolution, which would ‘divide’ the whole interacting network into several small parts. This leads to the occurrence of some small cliques of cooperators. As a result, *P*_*C*_ on scale-free networks under the upper-bound *W* = 4 is much lower than that on corresponding ring-4 network in all the cases.

**Fig 10:**
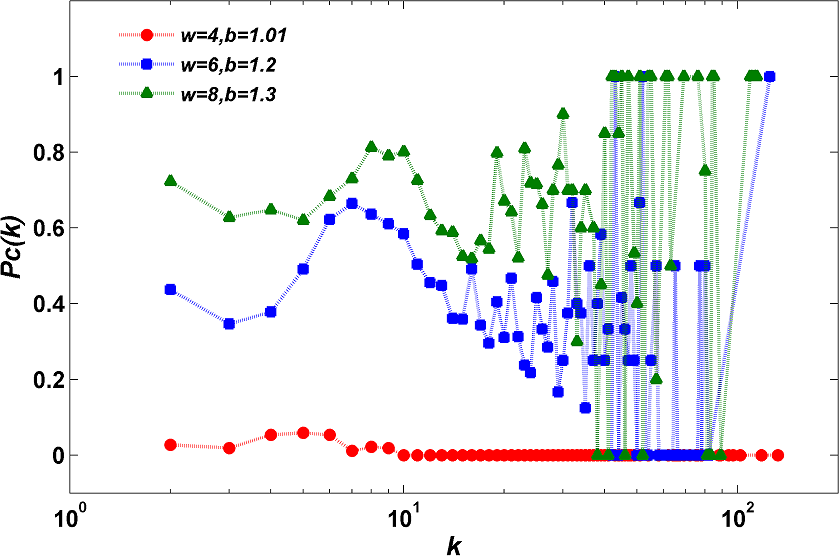
Probability *P*_*C*_(*k*) of a player of degree *k* playing as a cooperator in the stationary regime of evolutionary dynamics. The random selection rule is applied and *l* = 20.

Inversely, in Fig 8B and 8C, the higher payoffs of high-connectivity cooperators not only encourage their interacting-neighbors to cooperate because of the strategy updating rule defined by Eq (4), but also benefit cooperators to form a big cooperative cluster. It indicates that, the cooperators in the biggest connected sub-graph in the stationary regime of evolutionary dynamics account for 91.6% of all the cooperators under upper-bound *W* = 6, and 95.3% under upper-bound *W* = 8. As a result, the players with different degrees are more likely to cooperate under higher upper-bound *W* Fig 10 shows the probability *P*_*C*_(*k*) that a player of degree *k* acts as a cooperator in the stationary regime of evolutionary dynamics. For a given realization *l* of initial condition (*W*, *b*, selection rule), $P_{C}(k)=\sum_{l}n_{l}^{C}(k)/\sum_{l}n_{l}(k)$, where $n_{l}^{C}(k)$ is the number of cooperators of degree *k* and *n*_*l*_(*k*) is the number of players of that degree.

In Fig 10, we can observe that the players with different degrees are more likely to cooperate under higher upper-bounds. More specific data analysis results are provided as follows. For the players whose degrees *k* are between 2 and 32, a higher upper-bound *W* corresponds to a higher *P*_*C*_(*k*); for the players whose degrees *k* are larger than 32, a higher upper-bound *W* corresponds to a higher average level of *P*_*C*_(*k*), i.e., average *P*_*C*_(*k*) = 0 for *W* = 4, average *P*_*C*_(*k*) = 0.2721 for *W* = 6, and average *P*_*C*_(*k*) = 0.6495 for *W* = 8. These results indicate that under a higher upper-bound *W*, the players of all degrees are more resistant to defect temptation. As a result, the density of cooperators *P*_*C*_ on scale-free networks increases with upper-bound *W* and the interaction degree exhibits positive effect on cooperation on scale-free networks.

In brief, the relevant analysis from Figs 8 to 10 indicates that more interactions on scale-free network help high-connectivity players choose cooperative behavior and obtain higher game payoff than defective ones. This encourages their interacting-neighbors to cooperate, and benefit the cooperators to form a big cooperative cluster which is conductive to the evolution of cooperators. As a result, the players with different degrees are more likely to cooperate under higher upper-bound *W*. A certain number of interactions are necessary for scale-free networks to exhibit strong capability in facilitating cooperation.

## Conclusive remarks

In this paper, we study the evolutionary Prisoner’s Dilemma Game on scale-free networks with limited interaction. Since the interaction is limited, individuals have to selectively interact with others. Then, the effect of interaction style on cooperation is the first problem we focus on. For this purpose, we introduce a universally applicable interaction style in the real world, i.e., high-degree preference interaction. Such a preference interaction is rooted in obtaining more benefits from the individuals who have more connections and resources, and is compared with random interaction. It is found that high-degree preference interaction is less beneficial for the evolution of cooperation than random interaction, because high-connectivity players form an interacting core group and then it is easier for high-connectivity defectors to invade the core group.

Meanwhile, we investigate the effect of interaction degree on cooperation under two different interaction styles, and examine whether the overwhelming advantage of scale-free networks against regular ring networks still exist if limited interaction, instead of full interaction, is considered. It is discovered that cooperation is enhanced with the increase of interaction degree on scale-free networks regardless of the interaction style (e.g., high-degree preference or random), and if the interaction degree is very low, the cooperation level on scale-free networks is much lower than that on regular ring networks. This discovery is against the common belief that scale-free networks must be more beneficial for cooperation than the ring network. It can be attributed to the fact that for a very low interaction degree, although some low-connectivity cooperators obtain higher payoff than others, it is difficult for them to form a big cooperative cluster which is conductive to the evolution of cooperation. On the opposite, with the increase of interaction degree, high-connectivity players cooperate and obtain higher payoff than others, which not only influence their interacting-neighbors to cooperate, but also benefit the cooperators to form a big cooperative cluster. Thus, a certain number of interactions are necessary for scale-free networks to prompt a high level cooperation.

The above results provide insightful information for individual interactions in the real world. Firstly, social organizations should provide their members more chances to interact in scale-free networks, i.e., more connections should be consciously built between leaders and ordinary staff. At the same time, leaders should interact actively and consciously with ordinary staff. The actual situation in the real world is that leaders generally have more connections and interactions among themselves, which does not benefit a high-level cooperation. Network structure, interaction style, and interaction degree are all essential to facilitate a high level of cooperation in a social organization.

Based on the current results, we can also envision some important extension work in the future. Firstly, social diversity (i.e., heterogeneous players) might favor cooperation in PDG [7–9,38], then it would be interesting to investigate the possible impact of running the current simulation model with a heterogeneous player set. If the upper-bound *W* is also heterogeneous and is related to the degree of players, what happens to the cooperation on scale-free networks? Obviously, the results also depend on the interaction rules. Besides, Holme-Kim scale-free network [29], Xulvi-Brunet–Sokolov scale-free networks [32], hierarchical scale-free network [47], multilayer networks [48], dynamic networks [49] and various social dilemmas [50,51] have been applied to study the mechanism of cooperation among selfish individuals. It is valuable to explore whether the interaction style and degree also have impact on cooperation under other network patterns or social dilemmas. Lastly, in the real world, the interaction number of individuals generally changes with the time rather than keep constant. For example, while a strategy leads to a high payoff in interaction, individuals generally become more active in interaction and prefer that strategy. Then, if the interaction degree evolves along with the strategy, does the effect of the interaction styles on cooperation on scale-free networks change? The rule of changing interaction degree should be related to the game payoff of players, just as the rule of strategy updating. Those considerations will further advance the study of cooperation on networks.

## Supporting information

S1 Text The simulation framework for the model described in section 2. The symbol ‘%…’ is the annotation for the framework. The simulation code was implemented in Matlab (R2015b).
(DOCX)

## References

[1] Hammerstein P, editor. Genetic and Cultural Evolution of Cooperation. 1st, ed. Cambridge, MA: MIT Press; 2003.

[2] Nowak MA. Five rules for the evolution of cooperation. Science. 2006; 314(5805): 1560–1563. https://doi.org/10.1126/science.1133755 doi: 10.1126/science.1133755 17158317 10.1126/science.1133755 PMC3279745

[3] Szabo G, Fath G. Evolutionary games on graphs. Physics Reports. 2007; 446(4–6): 97–216. https://doi.org/10.1016/j.physrep.2007.04.004

[4] Nowak MA, May RM. Evolutionary games and spatial chaos. Nature. 1992; 359(6398): 826–829. https://doi.org/10.1038/359826a0

[5] Szabó G, Tőke C. Evolutionary prisoner’s dilemma game on a square lattice. Physical Review E. 1998; 58(1): 69–73. https://doi.org/10.1103/PhysRevE.58.69

[6] Szabó G, Vukov J, Szolnoki A. Phase diagrams for an evolutionary prisoner’s dilemma game on two-dimensional lattices. Physical Review E. 2005; 72(4): 047107 https://doi.org/10.1103/PhysRevE.72.047107 10.1103/PhysRevE.72.047107 16383580

[7] Perc M, Szolnoki A. Social diversity and promotion of cooperation in the spatial prisoner’s dilemma game. Physical Review E. 2008; 77(1):011904 https://doi.org/10.1103/PhysRevE.77.011904 10.1103/PhysRevE.77.011904 18351873

[8] Santos FC, Santos MD, Pacheco JM. Social diversity promotes the emergence of cooperation in public goods games. Nature. 2008; 454(7201): 213–216.https://doi.org/10.1038/nature06940 doi: 10.1038/nature06940 18615084 10.1038/nature06940

[9] Liming W, Wu F. Effects of empty sites on cooperation in the prisoner’s dilemma game based on social diversity. Discrete Dynamics in Nature and Society. 2014; (2014): 907052 https://doi.org/10.1155/2014/907052

[10] Perc M, Szolnoki A, Szabó G. Restricted connections among distinguished players support cooperation. Physical Review E. 2008;78(6): 066101 https://doi.org/10.1103/PhysRevE.78.066101 10.1103/PhysRevE.78.066101 19256899

[11] Szolnoki A, Szabó G. Cooperation enhanced by inhomogeneous activity of teaching for evolutionary Prisoner's Dilemma games. Europhysics Letters. 2007; 77(3): 30004 https://doi.org/10.1209/0295-5075/77/30004

[12] Killingback T, Doebeli M, Knowlton N. Variable investment, the continuous prisoner's dilemma, and the origin of cooperation. Proceedings of the Royal Society of London B: Biological Sciences. 1999; 266(1430): 1723–1728. https://doi.org/10.1098/rspb.1999.0838 10.1098/rspb.1999.0838 PMC1690197 10518320

[13] Xia CY, Meng XK, Wang Z. Heterogeneous coupling between interdependent lattices promotes the cooperation in the prisoner’s dilemma game. PloS ONE. 2015; 10(6): e0129542 https://doi.org/10.1371/journal.pone.0129542 doi: 10.1371/journal.pone.0129542 26102082 10.1371/journal.pone.0129542 PMC4477883

[14] Zhu C, Sun S, Wang J, Xia C. Role of population density and increasing neighborhood in the evolution of cooperation on diluted lattices. Physica A: Statistical Mechanics and its Applications. 2013; 392(24): 6353–6360. https://doi.org/10.1016/j.physa.2013.07.069

[15] Watts DJ, Strogatz SH. Collective dynamics of ‘small-world’ networks. Nature. 1998; 393(6684): 440–442. https://doi.org/10.1038/30918 10.1038/30918 9623998

[16] Watts DJ. Small worlds: the dynamics of networks between order and randomness. 1st, ed. Princeton, NJ: Princeton university press; 1999.

[17] Barabási AL, Albert R. Emergence of scaling in random networks. Science. 1999; 286(5439): 509–512. https://doi.org/10.1126/science.286.5439.509 10521342 10.1126/science.286.5439.509

[18] Santos FC, Rodrigues JF, Pacheco JM. Epidemic spreading and cooperation dynamics on homogeneous small-world networks. Physical Review E. 2005; 72(5): 056128 https://doi.org/10.1103/PhysRevE.72.056128 10.1103/PhysRevE.72.056128 16383709

[19] Wu ZX, Xu XJ, Chen Y, Wang YH. Spatial prisoner’s dilemma game with volunteering in Newman-Watts small-world networks. Physical Review E. 2005; 71(3): 037103 https://doi.org/10.1103/PhysRevE.71.037103 10.1103/PhysRevE.71.037103 15903637

[20] Seltzer N, Smirnov O. Degrees of Separation, Social Learning, and the Evolution of Cooperation in a Small-World Network. Journal of Artificial Societies and Social Simulation. 2015;18(4): 12 https://doi.org/10.18564/jasss.2851

[21] Santos FC, Pacheco JM. Scale-free networks provide a unifying framework for the emergence of cooperation. Physical Review Letters. 2005; 95(9): 098104 https://doi.org/10.1103/PhysRevLett.95.098104 doi: 10.1103/PhysRevLett.95.098104 16197256 10.1103/PhysRevLett.95.098104

[22] Santos FC, Pacheco JM, Lenaerts T. Evolutionary dynamics of social dilemmas in structured heterogeneous populations. Proceedings of the National Academy of Sciences. 2006; 103(9): 3490–3494.https://doi.org/10.1073/pnas.0508201103 10.1073/pnas.0508201103 PMC1413882 16484371

[23] Tsukamoto E, Shirayama S. Influence of the variance of degree distributions on the evolution of cooperation in complex networks. Physica A: Statistical Mechanics and its Applications.2010; 389(3): 577–586.https://doi.org/10.1016/j.physa.2009.10.002

[24] Newman MEJ. Assortative mixing in networks. Physical review letters. 2002; 89(20): 208701 https://doi.org/10.1103/PhysRevLett.89.208701 doi: 10.1103/PhysRevLett.89.208701 12443515 10.1103/PhysRevLett.89.208701

[25] Newman MEJ. Mixing patterns in networks. Physical Review E. 2003; 67(2): 026126 https://doi.org/10.1103/PhysRevE.67.026126 10.1103/PhysRevE.67.026126 12636767

[26] Girvan M, Newman MEJ. Community structure in social and biological networks. Proceedings of the national academy of sciences. 2002; 99(12): 7821–7826. https://doi.org/10.1073/pnas.122653799 10.1073/pnas.122653799 PMC122977 12060727

[27] Arenas A, Danon L, Díaz-Guilera A, Gleiser PM, Guimerá R. Community analysis in social networks. The European Physical Journal B. 2004; 38(2): 373–380. https://doi.org/10.1140/epjb/e2004-00130-1

[28] Chen YS, Lin H, Wu CX. Evolution of prisoner’s dilemma strategies on scale-free networks. Physica A: Statistical Mechanics and its Applications. 2007; 385 (1): 379–384. https://doi.org/10.1016/j.physa.2007.06.008

[29] Assenza S, Gómez-Gardeñes J, Latora V. Enhancement of cooperation in highly clustered scale-free networks. Physical Review E. 2008; 78(1): 017101 https://doi.org/10.1103/PhysRevE.78.017101 10.1103/PhysRevE.78.017101 18764081

[30] Cong R, Qiu YY, Chen XJ, Wang L. Robustness of cooperation on highly clustered Scale-Free networks. Chinese Physics Letters. 2010; 27(3): 030203 https://doi.org/10.1088/0256-307X/27/3/030203

[31] Chen XJ, Fu F, Wang L. Prisoner’s Dilemma on community networks. Physica A: Statistical Mechanics and its Applications. 2008; 378 (2): 512–518. https://doi.org/10.1016/j.physa.2006.12.024

[32] Rong Z, Li X, Wang X. Roles of mixing patterns in cooperation on a scale-free networked game. Physical Review E. 2007; 76 (2): 027101 https://doi.org/10.1103/PhysRevE.76.027101 10.1103/PhysRevE.76.027101 17930177

[33] Szolnoki A, Perc M, Danku Z. Towards effective payoffs in the prisoner’s dilemma game on scale-free networks. Physica A: Statistical Mechanics and its Applications. 2008;387 (8–9): 2075–2082. https://doi.org/10.1016/j.physa.2007.11.021

[34] Du WB, Cao XB, Hu MB. The effect of asymmetric payoff mechanism on evolutionary networked prisoner’s dilemma game. Physica A: Statistical Mechanics and its Applications. 2009; 388(24): 5005–5012. https://doi.org/10.1016/j.physa.2009.08.026

[35] Wu ZX, Guan JY, Xu XJ, Wang YH. Evolutionary prisoner's dilemma game on Barabási–Albert scale-free networks. Physica A: Statistical Mechanics and its Applications. 2007; 379(2): 672–680.https://doi.org/10.1016/j.physa.2007.02.085

[36] Du WB, Cao XB, Zhao L, Hu MB. Evolutionary games on scale-free networks with a preferential selection mechanism. Physica A: Statistical Mechanics and its Applications. 2009; 388(20): 4509–4514.https://doi.org/10.1016/j.physa.2009.07.012

[37] Perc M. Evolution of cooperation on scale-free networks subject to error and attack. New Journal of Physics. 2009; 11(3): 033027 https://doi.org/10.1088/1367-2630/11/3/033027

[38] Szolnoki A, Perc M, Szabó G. Diversity of reproduction rate supports cooperation in the prisoner's dilemma game on complex networks. The European Physical Journal B. 2008; 61(4): 505–509. https://doi.org/10.1140/epjb/e2008-00099-7

[39] Du WB, Zheng HR, Hu MB. Evolutionary prisoner’s dilemma game on weighted scale-free networks. Physica A: Statistical Mechanics and its Applications. 2008; 387(14): 3796–3800. https://doi.org/10.1016/j.physa.2008.02.036

[40] Buesser P, Tomassini M. Super cooperation in evolutionary games on correlated weighted networks. Physical Review E. 2012; 85(1): 016107 https://doi.org/10.1103/PhysRevE.85.016107 10.1103/PhysRevE.85.016107 22400625

[41] Szolnoki A, Perc M. Conformity enhances network reciprocity in evolutionary social dilemmas. Journal of the Royal Society Interface. 2015;12(103): 20141299 https://doi.org/10.1098/rsif.2014.1299 10.1098/rsif.2014.1299 PMC4305429 25540242

[42] Cardillo A, Gómez-Gardeñes J, Vilone D, Sánchez A. Co-evolution of strategies and update rules in the prisoner's dilemma game on complex networks. New Journal of Physics. 2010; 12(10): 103034 https://doi.org/10.1088/1367-2630/12/10/103034

[43] Liu RR, Rong Z, Jia CX, Wang BH. Effects of diverse inertia on scale-free–networked prisoner's dilemma games. Europhysics Letters. 2010; 91(2): 20002 https://doi.org/10.1209/0295-5075/91/20002

[44] Chen YS, Lin H, Wu CX. Evolution of prisoner's dilemma strategies on scale-free networks. Physica A: Statistical Mechanics and its Applications. 2007; 385(1): 379–384.https://doi.org/10.1016/j.physa.2007.06.008

[45] Szolnoki A, Xie NG, Ye Y, Perc M. Evolution of emotions on networks leads to the evolution of cooperation in social dilemmas. Physical Review E. 2013; 87(4): 042805 https://doi.org/10.1103/PhysRevE.87.042805 10.1103/PhysRevE.87.042805 23679471

[46] Poncela J, Gómez-Gardenes J, Moreno Y. Cooperation in scale-free networks with limited associative capacities. Physical Review E. 2011; 83(5): 057101 https://doi.org/10.1103/PhysRevE.83.057101 10.1103/PhysRevE.83.057101 21728697

[47] Li Y, Jin X, Kong F, Luo H. Strategic games on a hierarchical network model. Journal of Zhejiang University-Science A. 2008; 9(2): 271–278.https://doi.org/10.1631/jzus.A071331

[48] Wang Z, Wang L, Szolnoki A, Perc M. Evolutionary games on multilayer networks: a colloquium. The European Physical Journal B. 2015; 88: 124 https://doi.org/10.1140/epjb/e2015-60270-7

[49] Perc M, Szolnoki A. Coevolutionary games—a mini review. Biosystems. 2010; 99(2): 109–125. https://doi.org/10.1016/j.biosystems.2009.10.003 doi: 10.1016/j.biosystems.2009.10.003 19837129 10.1016/j.biosystems.2009.10.003

[50] Du WB, Cao XB, Hu MB, Wang WX. Asymmetric cost in snowdrift game on scale-free networks. Europhysics Letters. 2009; 87(6): 60004 https://doi.org/10.1209/0295-5075/87/60004

[51] Wang Z, Bauch CT, Bhattacharyya S, d'Onofrio A, Manfredi P, Perc M, et al Statistical physics of vaccination. Physics Reports. 2016; 664: 1–113. https://doi.org/10.1016/j.physrep.2016.10.006.

