# Supplementary material for: Impact of interaction style and degree on the evolution of cooperation on Barabási–Albert scale-free network
Source: PLoS One. 2017 Aug 14;12(8):e0182523. doi: 10.1371/journal.pone.0182523 (PMC5555699; doi:10.1371/journal.pone.0182523)
Supplement: S1 Text — The symbol ‘%…’ is the annotation for the framework. The simulation code was implemented in Matlab (R2015b). (DOCX) [file pone.0182523.s001.docx]

## Supporting information

The simulation framework for the model described in section 2 is presented as follows. The symbol ‘%....’ is the annotation for the framework. The simulation code was implemented in Matlab (R2015b).

| Initialization of parameter values ($N=1000,10000;m=m_{0}=2;R=1, P=S=0,2>b>1;W=4,or,6,8,12,16,32;t=11000$)  **for** a given initialization condition **do** *% Repeating experiments to obtain average value of results*  GENERATE a scale-free network by Barabási-Albert algorithm ***% (section 2.1)***  % Each node represents a game player |
| --- |
| ***% (section 2.2 begins)***  SORT for players according to their degrees  ASSIGN a strategy (C or D) randomly to each player  *% The initial strategies are randomly assigned among players with equal probability*  **for** each game time (*t*) **do**  *% Game time t begins* |
| **for** each player *i* **do** *% Each player selects interacting-neighbors sequentially* |
| **if** $n_{\Omega_{i,t}^{actual}}<W$  IDENTIFY player *i*’s neighbors who are not his interacting-neighbors  IDENTIFY player *i*’s neighbors whose $n_{\Omega_{j,t}^{actual}}<W$  **if** there is no any neighbor who is satisfied with the above two requirements  player *i does nothing in this selection*  **otherwise**  player *i* SELECTS interacting-neighbors according to a certain selection rules  *% high-degree selection rule according to* *formula (2), or, random selection rule*  **end**  **end**  **end** *% Interacting-neighbors selecting for game time t has been finished*  **for** each player *i* **do** *% Each player calculates game payoff* |
| **if** $n_{\Omega_{i,t}^{actual}}>0$ *% if player i has interacting-neighbor at game time t*  CALCULATE game payoff $U_{i,t}$ according to formula (3)  **else**  *%*  *if player i does not have interacting-neighbor at game time t* $U_{i,t}=0$   **end**  **end** *% Calculating game payoff has been finished*  **for** each player *i* **do** *% Each player updates strategy*  **if** $n_{\Omega_{i,t}^{actual}}>0$  player *i* UPDATEs strategy according to formula (6)  **else**  player *i* keeps strategy invarible  **end** |
| **end**  *% Updating strategy has been finished* |
| **end** *% Game time t ends, returns to blue ‘****for****...****do****’ and game time t+1 begins*  ***% (Section 2.2 ends)*** |
| **end** *% Repeating experiments end* |
